# Lignin peroxidase mediated silver nanoparticle synthesis in *Acinetobacter* sp.

**DOI:** 10.1186/s13568-017-0528-5

**Published:** 2017-12-22

**Authors:** Richa Singh, Utkarsha U. Shedbalkar, Shradhda B. Nadhe, Sweety A. Wadhwani, Balu A. Chopade

**Affiliations:** 1 Department of Microbiology, Savitribai Phule Pune University, Pune, 411007 India; 2 Department of Biochemistry, The Institute of Science, Mumbai, 400032 India; 3 Dr. Babasaheb Ambedkar Marathwada University, Aurangabad, 431004 India

**Keywords:** Silver nanoparticles, *Acinetobacter*, Lignin peroxidase, Purification, PAGE

## Abstract

Metals present in environment render the bacteria to attain certain resistance machinery to survive, one of which is transformation of metal ions to nano forms. Various enzymes and proteins have been suggested to play significant role in synthesis of silver nanoparticles (AgNPs) in bacteria. In present study, we have purified lignin peroxidase from secreted enzyme extract of *Acinetobacter* sp. employing diethyl aminoethyl cellulose ion exchange and Biogel P-150 gel filtration column chromatography. The purified fraction has a specific activity of 1.571 U/mg with substrate *n*-propanol and 6.5-fold purification. The tetrameric enzyme, with molecular weight of 99 kDa, consisted of dimers of two polypetides of 23.9 and 24.6 kDa as revealed by native and SDS-PAGE. On exposure to purified enzyme, spherical polydispersed AgNPs of ~ 50 nm were obtained as observed under transmission electron microscope. Optimum activity of the purified enzyme was obtained at pH 2 and 60 °C with *n*-propanol as substrate. This is the first report describing the reduction of extracellular silver ions by lignin peroxidase purified from *Acinetobacter* sp.

## Introduction

*Acinetobacter* species are aerobic, non-motile and Gram negative coccobacilliary bacteria (Huddedar et al. 2002; Pardesi et al. 2007; Singh et al. 2016; Towner and Chopade 1987). They are quite versatile with respect to genetic organization, nutrition and metabolism leading their ubiquitous presence in soil, water, food, industrial wastes, sludge, human skin, respiratory mucus membrane, etc. (Jagtap et al. 2010; Patil and Chopade 2000; Rokhbakhsh-Zamin et al. 2011; Sachdev et al. 2010; Saha and Chopade 2002; Yavankar et al. 2007; Yele et al. 2012). Bacteria encounter various metals and metalloids, such as copper, zinc, manganese, vanadium, chromium, molybdenum, silver, iron, cobalt, nickel, etc. in nature. Some of these ions are required for structural or catalytic functions in bacteria while others have detrimental effect (Deshpande et al. 1993; Shakibai et al. 1998, 1999). To thrive in such conditions, bacterium attains certain genetic and biochemical mechanisms, such as extracellular binding, precipitation, segregation and complexation by thiol-containing molecules, intracellular deposition, solubility variation and alteration in toxicity by changing redox state of the metal ion (Deshpande and Chopade 1994; Dhakephalkar and Chopade 1994; Singh et al. 2015). Absence of specific metal transporter system or any cellular efflux pumping systems has also been suggested to prevent metal ion deposition in lethal concentration inside the cells (Das and Marsili 2010). Moreover, transformation of metal ions to nanoparticles may also act in favor of microorganisms (Duran et al. 2011).

*Acinetobacter* strains are capable of synthesizing large number of metal nanoparticles, such as silver, gold, selenium, platinum, etc. (Gaidhani et al. 2013; Singh et al. 2013; Wadhwani et al. 2014, 2016). Reduction of silver ions to silver nanoparticles (AgNPs) is of particular interest for *Acinetobacter* spp. that are known to exhibit plasmid-mediated silver resistance (Deshpande and Chopade 1994). Moreover, *Acinetobacter* strains have also been reported to possess a cysteine-rich metalloprotein, which aid in binding and accumulating silver ions intracellularly without any toxicity (Shakibai et al. 2003). In our earlier study, we demonstrated the production of extracellular AgNPs employing secreted enzymes in *Acinetobacter* sp. (Singh et al. 2013). The available literature on bacteriagenic AgNPs suggested the role of NADH-dependent enzymes, especially nitrate reductase, in reduction of silver ions to nano silver (Kalimuthu et al. 2008; Karthik et al. 2014). However, *Acinetobacter* spp. do not produce nitrate reductase enzyme, the fact supported by the study of Gaidhani et al. (2013), where nitrate-reductase independent synthesis of AgNPs was reported. Nitrogenase and hydrogenase enzymes have been implicated in formation of silver nanoparticles in cyanobacteria (Brayner et al. 2007). Besides this, the reducing cofactors generated by spore-associated enzymes, such as alkaline phosphatase, glucose oxidase, catalase and laccase, can also mediate AgNP synthesis in bacteria (Hosseini-Abari et al. 2013). In one of the non-biological processes, conjugate of streptavidin–horseradish peroxidase (HRP) has been demonstrated to stimulate growth of AgNPs on a silicon surface (Schneidewind et al. 2012).

Since extract of secreted organic compounds by *Acinetobacter* sp. is capable of synthesizing extracellular AgNPs (Singh et al. 2013), we propose that this secretory material has vital proteins, including enzymes, and other biomolecules released by *Acinetobacter* sp. while growing. These organic molecules may act as reducing agents to convert silver ions to AgNPs. In view of this, the present study deals with the purification and biochemical characterization of one of the proteins involved in AgNP synthesis in *Acinetobacter* sp. The purified protein was evaluated for its potential activity in reduction of silver ions to AgNPs.

## Materials and methods

### Chemicals

Luria–Bertani (LB) broth, agar, catechol, veratryl alcohol, magnesium sulfate, zinc sulfate, copper sulfate, ammonium sulfate, silver chloride, mercuric chloride, calcium chloride, sodium chloride, dipotassium hydrogen phosphate, potassium dihydrogen phosphate, silver nitrate, sodium nitrate, potassium nitrate, ammonium nitrate and sodium thiosulfate, sulfanilamide, *N*-(1-naphthyl)ethylenediamine dihydrochloride (NEDD), 2,2′-Azino-bis(3-ethylbenzothiazoline-6-sulfonic acid) diammonium salt (ABTS), Diethyl aminoethyl (DEAE)-cellulose and Biogel P-150 were procured from Himedia (Mumbai, India). Coomassie brilliant blue R-250, hydrogen peroxide and *n*-propanol were obtained from Loba Chemie (Mumbai, India), SD Fine (Mumbai, India) and Qualigens (Mumbai, India), respectively. Ammonium sulphate, NADH, L-DOPA, guaiacol, hydroxyquinolone and tartaric acid were supplied by SRL Pvt Ltd (Mumbai, India).

### Preparation of secreted enzyme extract

*Acinetobacter* sp. (MCC 3391), isolated from wheat rhizosphere, was routinely cultured in LB broth. Prior to inoculation for experiments, a loopful of culture from LB agar plate was inoculated in 10 ml LB broth in tube and incubated overnight at 37 °C/150 rpm to obtain log-phase metabolically active bacterial cells.

The secreted enzyme (SE) extract of 72 h cultivation, reported to produce silver nanoparticles, has been prepared as per the protocol described in our earlier study (Singh et al. 2013). Briefly, OD adjusted (OD_600_ ~ 1.0) overnight grown log-phase culture of *Acinetobacter* sp. was inoculated in 250 ml flask containing 100 ml LB broth (1% v/v) and incubated at 30 °C/150 rpm for 24 h. The cells were harvested by centrifugation and resuspended in sterile distilled water after thorough washing. These were then incubated at 30 °C/150 rpm. After 72 h, centrifugation was done and cell pellet was discarded. SE extract was collected by passing the supernatant obtained after centrifugation through a 0.2 μm membrane filter (Pall Corporation, NY, USA) and stored at 4 °C until further use.

### Protein estimation and enzyme activity

Protein was determined by Folin–Lowry method (Lowry et al. 1951), using bovine serum albumin as a standard.

Nitrate reductase activity in SE extract was determined by incubating the extract with 10 mM potassium nitrate and 2 mM NADH for 2 min at 30 °C followed by addition of 50 mM sulphanilamide (prepared in 3 M hydrochloric acid) and 77 mM NEDD. The positive nitrate reductase activity was detected by presence of pink color, the absorbance of which was measured at 540 nm. Suitable blanks were also maintained.

Laccase enzyme activity was determined using 0.1% ABTS as a substrate in acetate buffer (0.1 M, pH 4). One unit of enzyme activity was defined as the amount of enzyme required to oxidize 1 μmol of substrate in 1 min, which was measured by reading absorbance at 420 nm for 1 min.

Lignin peroxidase activity was measured using *n*-propanol as a substrate as per the protocol described earlier (Kalyani et al. 2011). The reaction mixture contained 40 mM *n*-propanol, 2 mM hydrogen peroxide and 50 mM tartaric acid. One unit of enzyme activity was defined as the amount of enzyme required to oxidize 1 μmol of substrate per minute. It is measured by reading absorbance at 310 nm for 1 min.

### Preparation of crude enzyme

The SE extract was subjected to ammonium sulphate precipitation to obtain 60% saturation in cold conditions. The solution was then centrifuged to collect the precipitates, which were further dialyzed against phosphate buffer (pH 7.8) to remove ammonium sulphate.

### Purification of enzyme

#### DEAE-cellulose ion exchange chromatography

The dialyzed crude preparation was loaded onto a preactivated DEAE-cellulose column (15 × 2 cm) equilibrated with 50 mM phosphate buffer (pH 7.8). The column was washed with same buffer and further elution was carried out using linear gradient of 0.2 to 1.0 M NaCl solution. Fractions containing lignin peroxidase activity were dialyzed against distilled water.

#### Biogel P-150 gel filtration chromatography

The dialyzed fraction was concentrated to 1 ml in sucrose and loaded on a Biogel P-150 column (45 × 0.8 cm) equilibrated with phosphate buffer (pH 7.8) and eluted with same buffer at a flow rate of 3 ml/h. Fractions containing lignin peroxidase activity were pooled, dialyzed against distilled water and stored at 4 °C until further use.

### Polyacrylamide gel electrophoresis (PAGE)

Native and sodium dodecyl sulphate (SDS)-PAGE was performed with 4% stacking and 10% resolving gel using vertical gel electrophoresis system (GeNei, Bangalore, India). Silver staining was performed in native PAGE and molecular mass of the purified peroxidase was determined by calculating the relative mobility of standard protein molecular weight marker (GeNei) run alongside. In SDS PAGE, protein bands were stained with 0.1% (w/v) coomassie brilliant blue R-250 prepared in methanol:acetic acid:distilled water (v/v) (4:1:5) for 3 h at room temperature. The gel was further destained with solution containing methanol:acetic acid:distilled water (v/v) (4:1:5) and the bands were analyzed with respect to the standard protein molecular weight marker (Sigma).

### AgNP synthesis by purified protein

Synthesis of AgNP by purified enzyme was carried out by addition of 0.07 mg enzyme, having 0.1 U of lignin peroxidase activity, to 1 mM silver nitrate solution in total reaction volume of 1 ml. The spectrum was measured over the wavelength range of 200–800 nm after regular intervals till 24 h. It was further analyzed under transmission electron microscope (TEM), dynamic light scattering (DLS) and energy dispersive spectroscopy (EDS) for morphological and compositional characteristics.

### Determination of optimum pH and temperature

The effect of pH on activity of 0.1 U of purified enzyme was assayed within a pH range of 1–10 using 40 mM *n*-propanol as a substrate and 50 mM of the following buffers: HCl–KCl buffer (pH 1), tartaric acid (pH 2), citrate buffer (pH 3), acetate buffer (pH 4–5), phosphate buffer (pH 6–8), tris–HCl buffer (pH 9) and carbonate–bicarbonate buffer (pH 10). Optimum temperature of the enzyme (0.1 U) was determined over the temperature range of 10–90 °C with 40 mM *n*-propanol as substrate at its optimal pH value. The experiments were performed in duplicate and the values are expressed as mean ± standard deviation.

### Effect of salts

Fifteen salts, including magnesium sulfate, zinc sulfate, copper sulfate, ammonium sulfate, silver chloride, mercuric chloride, calcium chloride, sodium chloride, dipotassium hydrogen phosphate, potassium dihydrogen phosphate, silver nitrate, sodium nitrate, potassium nitrate, ammonium nitrate and sodium thiosulfate, were added to the reaction mixture to obtain final concentration of 25 mM. Lignin peroxidase activity (0.1 U) was determined using 40 mM *n*-propanol as substrate using the protocol described earlier.

### Substrate specificity

Substrate specificity of peroxidase was checked by using 40 mM of various substrates, such as *n*-propanol, L-DOPA, guaiacol, hydroxyquinone, catechol and veratryl alcohol, prepared in double distilled water. The specific activity of enzyme was estimated through spectroscopic measurement of oxidation of substrates by enzyme at specific wavelength.

## Results

### Enzyme activity in SE extract

Previously, we showed extracellular synthesis of AgNPs employing SE extract of *Acinetobacter* sp. MCC 3391 (Singh et al. 2013). In present study, the bacterial SE extract was tested for presence of nitrate reductase, laccase and lignin peroxidase. SE extract showed positive activity for lignin peroxidase. However, no nitrate reductase and laccase activity was detected.

### Purification of lignin peroxidase

**Fig. 1:**
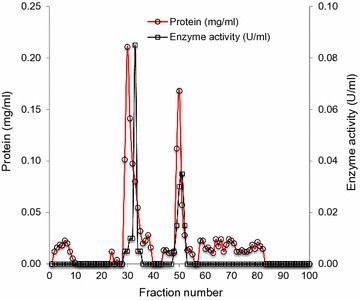
Elution profile of lignin peroxidase by DEAE-cellulose ion exchange chromatography

**Fig. 2:**
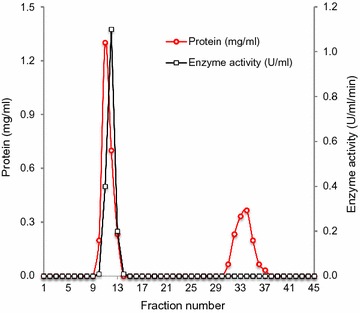
Elution profile of lignin peroxidase by Biogel P-150 gel filtration chromatography

To investigate the role of bacterial lignin peroxidase, present in SE extract, in synthesis of AgNPs, the enzyme was purified in two steps using column chromatography. Figure 1 represents the elution profile of the crude enzyme loaded on DEAE-cellulose anion exchanger column where linear gradient of 0.2–1.0 M NaCl was used as eluent. Although both fraction nos. 33 and 51 exhibited lignin peroxidase activity, fraction no. 33 had the higher specific activity and synthesized AgNPs faster as compared to fraction no. 51. Fraction no. 33 was further concentrated and loaded on Biogel P-150 gel filtration column. The elution profile has been shown in Fig. 2. Here, fraction no. 12 showed the lignin peroxidase activity.

Table 1 summarizes the purification profile of the enzyme. The crude enzyme contained a total of 165 mg protein corresponding to 40 U of bacterial lignin peroxidase activity with *n*-propanol as substrate. Further with two-step purification by DEAE-cellulose anion exchanger and Biogel P-150 gel filtration chromatography, the fraction with 6.5-fold increased peroxidase activity was obtained having protein concentration of 0.7 mg/ml and specific activity of 1.571 U/mg.

**Table 1:** Purification profile of lignin peroxidase from SE extract of *Acinetobacter* sp.

Purification stage	Enzyme activity (U)	Total protein (mg)	Specific activity (U/mg)	Purification fold
Crude	40	165	0.242	1.0
DEAE-cellulose anion exchanger	6.35	6.07	1.062	4.4
Biogel P-150 gel filtration	1.10	0.70	1.571	6.5

### PAGE

**Fig. 3:**
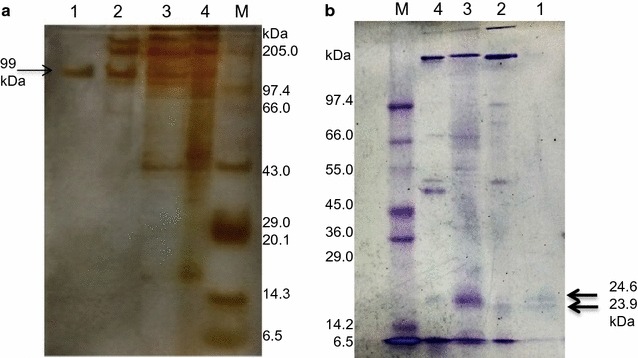
PAGE of purified enzyme fraction. **a** Native PAGE, and **b** SDS-PAGE. Lanes: 1 Biogel fraction, 2 DEAE-cellulose fraction, 3 ammonium sulphate precipitation (crude enzyme), 4 SE extract and M marker

The fraction no. 12, as obtained by gel filtration chromatography, showed a single band on native-PAGE stained by silver. The molecular mass of the purified fraction was 99 kDa (Fig. 3a). Further, the fraction was subjected to denaturation and analyzed on SDS-PAGE. Two bands, corresponding to molecular mass of 23.9 and 24.6 kDa, were seen (Fig. 3b).

### AgNP synthesis by purified lignin peroxidase

**Fig. 4:**
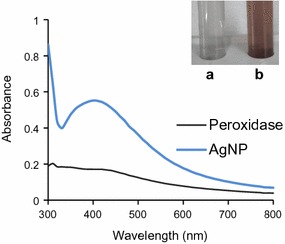
UV–Vis spectrum of purified lignin peroxidase and synthesized AgNPs. Inset: Color change showing AgNP synthesis by purified enzyme on addition of silver nitrate **a** control, and **b** after AgNP synthesis

**Fig. 5:**
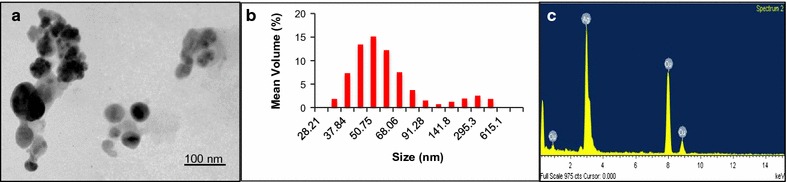
Characterization of AgNPs synthesized by purified lignin peroxidase by **a** TEM, **b** particle size distribution, and **c** EDS

Synthesis of AgNPs by purified lignin peroxidase was carried out. Exposure of purified lignin peroxidase to silver nitrate resulted in synthesis of AgNPs indicated by color change of the reaction medium to brown (Fig. 4). This change in color was observed within 6 h. An SPR peak at 400 nm was also observed in UV–Vis spectrum taken after 24 h confirming the conversion of silver ions to AgNPs (Fig. 4). No further increase in peak was observed after 24 h indicating complete reduction of silver ions to nanoparticles. Further, polydispersed spheres of nanoparticles of size ~ 50 nm were observed under TEM (Fig. 5a). The nanoparticles were seen to be present in both dispersed and aggregated forms, which was also confirmed by particle size distribution (Fig. 5b). There was no apparent effect of time of incubation on morphology of nanoparticles. EDS spectrum revealed a peak at 3 keV (Fig. 5c).

### Optimum pH and temperature

**Fig. 6:**
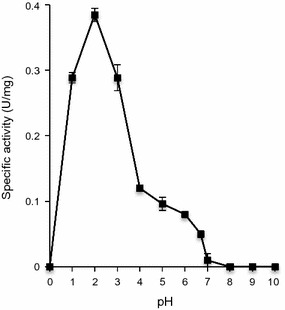
Optimum pH for lignin peroxidase with *n*-propanol as substrate. The experiment was done in duplicate and the values are expressed as mean ± SD

**Fig. 7:**
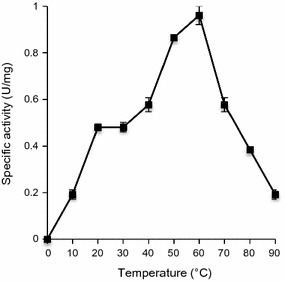
Optimum temperature for lignin peroxidase with *n*-propanol as substrate. The experiment was done in duplicate and the values are expressed as mean ± SD

The pH optimum of purified lignin peroxidase that catalyzed *n*-propanol oxidation was found to be 2.0 (Fig. 6). The specific activity decreased as the pH fell in alkaline range. The lignin peroxidase activity was observed at the temperature ranging from 10 to 90 °C indicating the great thermostability of the enzyme (Fig. 7). The optimum temperature with *n*-propanol was found to be 60 °C.

### Effect of salts

Effect of various salts on the activity of purified lignin peroxidase was tested with *n*-propanol as substrate (Table 2). Maximum enhancement in peroxidase activity was observed with magnesium sulfate followed closely by silver nitrate. These salts appear to act as good activators of enzyme activity. In general, addition of sulfate and nitrate ions increases the activity of the enzyme except when they are present in conjunction with ammonium ions. Ammonium salts, such as ammonium nitrate and ammonium sulfate, were found to inhibit the peroxidase activity. Chloride and phosphate salts also reduced the activity of the enzyme to some extent.

**Table 2:** Effect of salts on activity of purified enzyme

Salts	Specific activity (U/mg)
Control (without salt)	1.570
Magnesium sulfate	1.982
Zinc sulfate	1.040
Copper sulfate	1.648
Ammonium sulfate	0.127
Silver chloride	1.400
Mercuric chloride	1.400
Calcium chloride	1.400
Sodium chloride	1.400
Silver nitrate	1.451
Sodium nitrate	1.756
Potassium nitrate	1.410
Ammonium nitrate	0.062
Sodium thiosulfate	1.800
Dipotassium hydrogen phosphate	1.206
Potassium dihydrogen phosphate	1.217

### Substrate specificity

Substrate specificity of the purified lignin peroxidase was determined with various substrates (Table 3). The activity was found to be maximum with *n*-propanol closely followed by guaiacol, catechol and veratryl alcohol. No activity was detected with hydroxyquinone.

**Table 3:** Substrate specificity of purified lignin peroxidase

Substrates	Specific activity (U/mg)
*n*-Propanol	1.571
Catechol	1.361
Guaiacol	1.426
Hydroxyquinone	ND
Veratryl alcohol	1.127

*ND* not detected

## Discussion

Bacterial enzymes play an important role in transformation of metal ions to nanoparticles. Nitrate reductase, amylase, urease, aspartate protease and laccase have been demonstrated to synthesize silver, gold, copper, platinum and other metal nanoparticles (Duran et al. 2015; Reith et al. 2012; Sanghi et al. 2011; Shedbalkar et al. 2014). In present study, we have purified and characterized the enzyme responsible for extracellular AgNP synthesis in *Acinetobacter* sp. MCC 3391. Positive lignin peroxidase activity in SE extract confirmed that the bacteria secrete enzymes and other biomolecules extracellularly, which may act as reducing agent to convert silver ions to AgNPs. Lignin peroxidase is present mainly in fungi, such as *Phanerochaete chrysosporium*, *Gloeophyllum sepiarium* and *Cladosporium herbarum* and acts to degrade lignin (Yadav et al. 2010; Wang et al. 2008). However, few bacteria, such as *Acinetobacter calcoaceticus, Brevibacillus laterosporus* and *Streptomyces viridosporus*, are also known to produce lignin peroxidase (Ghodake et al. 2009; Gomare et al. 2008; Nascimento and Silva 2008). Our results, with respect to presence of nitrate reductase in *Acinetobacter* sp., is in accordance with the study of Gaidhani et al. (2013), where nitrate reductase independent synthesis of AgNPs was reported.

Employing two-step purification of the enzyme present in SE extract, through DEAE-cellulose anion exchanger and Biogel P-150 gel filtration column chromatography, 6.5-fold purified fraction was obtained with specific activity of 1.571 U/mg. Our results showed lower purification as compared to Ghodake et al. (2009), where 72-fold purification was reported employing only DEAE-cellulose chromatography. In a similar study, Kalyani et al. (2011) reported 17.1-fold purification for peroxidase enzyme from *Pseudomonas* sp. SUK1. Single protein band of the fraction observed on native-PAGE confirmed the purity of the fraction whereas two bands on SDS-PAGE indicated the tetrameric nature of lignin peroxidase in which two dimers, having molecular weight of approximately 47.8 (= 2 × 23.9) and 49.2 (= 2 × 24.6) kDa, are joined together. Dimeric nature of lignin peroxidase in *A. calcoaceticus* NCIM 2890 has been suggested earlier, where the molecular mass of the enzyme was reported to be 110 kDa (Ghodake et al. 2009). Other reports on bacterial peroxidases purified from *Pseudomonas* sp. SUK1 (Kalyani et al. 2011), *Bacillus* sp. VUS (Dawkar et al. 2009) and *B. laterosporus* MTCC 2298 (Gomare et al. 2008) showed molecular weights of 86, 43 and 205 kDa, respectively. On contrary, lignin peroxidase of *Streptomyces* sp. is a small protein of only 18 kDa (Nascimento and Silva 2008).

Preliminary testing of AgNP synthesis by purified enzyme was done by visual observation. The change in color of the solution from colorless to reddish brown is well known for reduction of silver ions to AgNPs (Singh et al. 2013; Gaidhani et al. 2013). Besides this, a single SPR peak located between 400 and 440 nm indicates the formation of spherical nanoparticles of size ≤ 100 nm (Henglein 1993), as also confirmed by TEM and particle size distribution. According to Mie’s theory, spherical metal nanoparticles produce single SPR peak while anisotropic particles, depending upon the shape, could give rise to two or more such bands (He et al. 2002; Novak and Feldheim 2000). In EDS spectrum, a peak at 3 keV is typical for absorption of AgNPs due to SPR (Singh et al. 2013), thereby confirmed the transformation of silver ions to nano silver through purified lignin peroxidase enzyme. Majority of reports suggested the involvement of NADH and NADH-dependent nitrate reductase in bacteria mediated silver nanoparticle synthesis (Chauhan et al. 2013; Fayaz et al. 2011; Kalimuthu et al. 2008; Karthik et al. 2014; Srivastava et al. 2013). Moreover, Kumar et al. (2007) demonstrated the in vitro synthesis of AgNPs of size 10–25 nm employing pure α-NADPH-dependent nitrate reductase. Besides this, other enzymes, such as cysteine desulfhydrase (C–S lyase), NfsA nitroreductase, oxidoreductase and phytochelatin synthase has also been implicated in reduction of metal ions to metal nanoparticles in microorganisms (Bai et al. 2011; Jha and Prasad 2010; Mohanpuria et al. 2008; Shahverdi et al. 2007). In one study, synthesis of enzymatically grown AgNPs has also been demonstrated, where a streptavidin–horseradish peroxidase (HRP) conjugate, bound to the silicon surface via DNA linker molecule, catalyzed the silver ion reduction (Schneidewind et al. 2012). However, this is the first study in *Acinetobacter* spp. to elucidate the involvement lignin peroxidase, secreted extracellularly, in transformation of silver ions.

Lignin peroxidase belongs to oxidoreductase family of enzymes, which does not require cofactors like NADH or NADPH. Here, the nanoparticle synthesis was obtained without addition of any peroxide derivative. This could be due to the presence of free cysteine in the enzyme. According to Glumoff et al. (1990), most lignin peroxidases possess five cysteine residues having sulfhydryl groups that may act as reducing agent. Besides this, hydroxyl group in tyrosine residue may also donate electron for reduction of silver ions to AgNPs. Sulfhydryl group in cysteine of laccase and urease enzymes, hydroxyl groups in tyrosine and amine groups in tryptophan residues of lysozyme have been suggested to play a significant role in formation of AgNPs through reduction of silver ions (Duran et al. 2014; Kumar et al. 2012; Sharma et al. 2013). Generation of nanosilver through functional groups of amino acid residues may affect the activity of the enzyme as also indicated by decrease in its specific activity on exposure to silver nitrate (Table 2). On contrary to our results, Rangnekar et al. (1997) described the amylase-mediated generation of gold nanoparticles through Au–S bonds with retention of enzyme activity. However, complete elucidation of mechanism warrants further investigation.

The optimum pH purified enzyme that catalyzed *n*-propanol oxidation was found to be 2, which is in accordance with the study of Dawkar et al. (2009). Peroxidase purified from *Pseudomonas* sp. and *Bacillus* sp. also exhibited maximum activity in acidic pH close to 3.0 (Kalyani et al. 2011). However, as reported earlier, AgNP synthesis occurs in SE extract at pH 7 where AgNO_3_ acts as a substrate (Singh et al. 2013). This indicates that pH optimum and hence, the enzyme activity is dependent upon the substrate. Variation in pH of the reaction medium cause changes in the ionic forms of the active sites, which may result in change in enzyme activity and their three-dimensional structure (Hossain and Ananthraman 2006; Shulter and Kargi 2000). Moreover, in SE extract there are various other organic moieties, which help the enzyme to reduce silver ions to nanoparticles at neutral pH. In a similar finding, Ghodake et al. (2009) reported the optimum pH of lignin peroxidase enzyme to be 1 with *n*-propanol as substrate while dye decolorization by the enzyme occurred at pH 7.

Lignin peroxidase activity with substrate *n*-propanol was observed at a broad range of temperature from 10 to 90 °C signifying its excellent thermostability. The optimum temperature for AgNP synthesis using bacterial SE extract was 70 °C (Singh et al. 2013). In view of these facts, it is clear that the purified enzyme possess the ability to catalyze the reactions at high temperature and can be one of the proteins involved in AgNP synthesis from silver ions. Similar broad range temperature stability has been reported for lignin peroxidase enzymes purified from *A. calcoaceticus* NCIM 2890 (Ghodake et al. 2009) and *B. laterosporus* MTCC 2298 (Gomare et al. 2008). However, in contrary to our results, Kalyani et al. (2011) showed the maximum peroxidase activity at 40 °C, which reduced drastically at temperature above 40 °C. Fungal lignin peroxidases exhibit temperature optimum in the range of 23–25 °C (Wang et al. 2008). In accordance with our study, magnesium sulfate and ammonium nitrate has been reported to act as lignin peroxidase activator and inhibitor, respectively (Kalyani et al. 2011). Manganese ions have also been reported to enhance the production of lignin peroxidase in *Phanerochaete chrysosporium* (Wang et al. 2008). Microbial peroxidases have wide substrate specificity and can easily interact with hydroxyl and methoxy-substituted phenols (Kim and Shoda 1999; Yang et al. 2005). Lignin peroxidase from *P. chrysosporium* showed maximum activity with ferrocyanide followed by *N*,*N*-dimethyl-1,4-phenylenediamine, anisyl alcohol, veratryl alcohol and guaiacol (Glumoff et al. 1990). Purified lignin peroxidase, in our study, also exhibited broad substrate specificity. However, the results are contradictory where we obtained higher activity with guaiacol than veratryl alcohol. It is well documented that lignin peroxidase in fungi degrades lignin with involvement of veratryl alcohol, which is a secondary metabolite and acts as a cofactor for the enzyme (Wang et al. 2008).

To summarize, we have purified lignin peroxidase from SE extract of *Acinetobacter* sp. and demonstrated its the involvement in synthesis of extracellular polydispersed spherical AgNP of ~ 50 nm. The purified enzyme is a 99 kDa protein consisting of two dimers and showed the optimum pH and temperature activity at 2 and 60 °C with *n*-propanol as the favored substrate. Moreover, magnesium sulfate and silver nitrate act as activators of the enzyme while ammonium salts inhibited its activity. It is important to note that SE extract contains numerous organic molecules, which can act to reduce silver ions to AgNPs and lignin peroxidase is one of them. This explains the variation in size and dispersion observed during synthesis using SE extract and purified enzyme. Further characterization and sequencing of the purified lignin peroxidase should be done by mass spectrometry, which can be used for in silico studies. This study further enables designing rational strategy for enzymatic approach of rapid and effective nanoparticle synthesis with novel morphologies, surface properties and unique functions.

## Abbreviations

AgNPs: silver nanoparticles
HRP: horseradish peroxidase
TEM: transmission electron microscope
EDS: energy dispersion spectroscopy
PAGE: polyacrylamide gel electrophoresis
NAD: nicotinamide adenine dinucleotide
NEDD: *N*-(1-naphthyl)ethylenediamine dihydrochloride
SE: secreted enzymes
SPR: surface plasmon resonance
